# „Crack lung“ – atypische bilaterale Pneumonie

**DOI:** 10.1007/s00108-024-01668-5

**Published:** 2024-02-16

**Authors:** Pia Maria Plank, Christopher Alexander Hinze, René Abu Isneineh, Hendrik Suhling

**Affiliations:** 1 Klinik für Kardiologie und Angiologie, Medizinische Hochschule Hannover, Carl-Neuberg-Straße 1, 30625 Hannover, Deutschland; 2 Klinik für Pneumologie und Infektiologie, Medizinische Hochschule Hannover, Hannover, Deutschland; 3 Klinik für Gastroenterologie, Medizinische Hochschule Hannover, Hannover, Deutschland

**Keywords:** Drogenabusus, Pneumonie, Respiratorische Insuffizienz, Pulmonale Hämorrhagien, Atypische Pneumonie, Drug abuse, Pneumonia, Respiratory insufficiency, Pulmonary hemorrhage, Atypical pneumonia

## Abstract

Die Inhalation von weiter verarbeitetem Kokain, wie *Crack* oder *Freebase*, kann zu alveolären Hämorrhagien führen. Das klinische Bild wird als „crack lung syndrome“ bezeichnet. Schwere Verläufe sind durch Zeichen einer progredienten hypoxämischen Insuffizienz mit Ausbildung eines akuten Lungenversagens (Acute Respiratory Distress Syndrom, ARDS) gekennzeichnet. In der Computertomographie des Thorax imponieren bipulmonale Konsolidierungen und Milchglasveränderungen mit oft subpleuralen Aussparungen. Unser Fallbericht eines 48-jährigen Patienten zeigt auf, dass neben dem Ausschluss infektiöser Ursachen die sorgfältige Anamnese zur Diagnosesicherung essenziell ist.

## Abstract (en)

Inhalation of crack and freebase results in alveolar hemorrhage. In severe courses of the disease, progressive respiratory insufficiency may lead to respiratory failure and acute respiratory distress syndrome (ARDS). Computed tomography of the thorax reveals bilateral consolidation and ground-glass pattern leaving a subpleural gap. This case report of a 48-year-old male patient highlights the importance of a thorough medical history while ruling out infectious causes.

## Anamnese

Ein 48-jähriger Mann stellte sich mit einem seit zwei Wochen zunehmenden Symptomkomplex aus Belastungsdyspnoe (schon bei leichter körperlicher Anstrengung), nichtproduktivem Husten, ungewolltem Gewichtsverlust, intermittierendem Fieber und Nachtschweiß in der Notaufnahme vor. Es imponierte eine periphere Sauerstoffsättigung (S_p_O_2_) von 88 % unter Raumluft. Die übrigen Vitalparameter waren im Normbereich. An relevanten Vorerkrankungen bestanden ein Asthma bronchiale und eine koronare Herzerkrankung. Der Patient berichtete von einem langjährig fortgeführten inhalativen Zigarettenrauchen und erst auf gezielte Nachfrage von einem gelegentlichen Cannabis- und Kokainkonsum sowie regelmäßigen Alkoholkonsum.

## Klinische Untersuchungsbefunde

Neben einem adipösen Ernährungszustand (BMI 31 kg/m^2^) konnten über beiden Lungenfeldern grobblasige Rasselgeräusche auskultiert werden. Hinzu kam eine beginnende periphere Zyanose mit gräulich-fahlem Integument und einer verlängerten Rekapillarisierungszeit. Hauteffloreszenzen, Lymphadenopathie und Gelenkschwellungen fanden sich nicht.

## Laborbefund

Im Labor zeigten sich eine Leukozytose (14,6 Tsd./µl) sowie ein erhöhtes CRP (187,4 mg/l), PCT (0,4 μg/l) und LDH (308 U/l). Die venöse Blutgasanalyse bei Aufnahme zeigte einen ausgeglichenen Säure-Basen-Haushalt mit Normokapnie.

Die erweiterte Labordiagnostik erbrachte ein erhöhtes IgE von 725 IU/ml (Normwerte bis 100 IU/ml) und eine erhöhte Gesamtkomplementaktivität der CH50 mit 59,8 U/ml (Normwert 31,6–57,6 U/ml). Autoantikörperbestimmungen (ENA, ANA, c‑ANCA, p‑ANCA, Anti-PR3, Anti-MPO, Anti-GBM, Anti-dsDNA) lieferten negative Befundergebnisse.

Im Drogenscreening waren Morphin/Derivate und Kokainmetaboliten positiv.

## Röntgenaufnahme des Thorax in 2 Ebenen

**Abb. 1:**
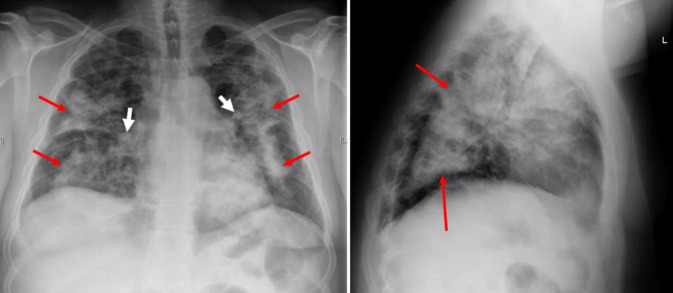
Röntgenuntersuchung des Thorax in 2 Ebenen

Diese zeigte ausgedehnte bipulmonale, teils flächige Transparenzminderungen peribronchovaskulär (*rote Pfeile*) mit subpleuraler Aussparung, vereinbar mit Infiltraten, sowie deutliche Bronchialwandverdickungen (*weiße Pfeile*; Abb. 1).

## Computertomographie des Thorax, nativ

**Abb. 2:**
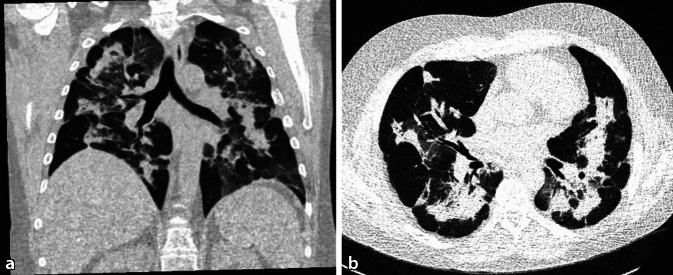
Computertomographie des Thorax nativ, **a** koronare und **b** transversale Ebene

Die Computertomographie zeigte seitensymmetrische bipulmonale Konsolidierungen und Milchglasveränderungen in sämtlichen Lungenlappen, vor allem in den zentralen Zonen mit überwiegender Aussparung des subpleuralen Raums (Abb. 2).

## Befunde

Es erfolgte eine Bronchoskopie mit bronchoalveolärer Lavage (BAL), mit zunehmender Rotfärbung der Spülfraktionen. Aus der BAL waren keine respiratorischen Viren nachweisbar, die Direkt-DNA-Nachweise von *Pneumocystis jirovecii* (PcP), *Chlamydia pneumoniae und psittaci, Mycoplasma pneumoniae *und *Legionella pneumophila* waren negativ. Mykobakterien oder Pilze waren in der BAL nicht nachweisbar.

Im Verlauf wurde aus dem Bronchialsekret vereinzelt *Klebsiella oxytoca* kulturell identifiziert.

Seriell abgenommene Blutkulturen erbrachten keinen Keimnachweis. Serologisch konnten eine Hepatitis (A, B, C, D), sowie HIV-1/2 ausgeschlossen werden.

Die Differenzialzytologie der BAL erbrachte eine mittelschwere Neutrophilie und keinen Hinweis auf PcP im Direktpräparat. Histopathologisch imponierte eine granulozytäre Entzündung mit minimaler Eosinophilie.

## Therapie und Verlauf

In der Notaufnahme wurde bei Verdacht auf eine ambulant erworbene Pneumonie eine kalkulierte antibiotische Therapie mit Ampicillin/Sulbactam und Azithromycin begonnen. Zu dem Zeitpunkt bestand ein Sauerstoffbedarf von 2 l über die Nasenbrille. Mittels Bronchoskopie und bronchoalveolärer Lavage konnten atypische Pneumonieerreger nicht nachgewiesen werden. Der klinische Verlauf wurde durch eine rasche hypoxämische Insuffizienz dominiert, sodass binnen weniger Stunden nach stationärer Aufnahme eine High-flow-Therapie begonnen wurde. Im Verlauf erschöpfte sich der Patient respiratorisch zunehmend, das CO_2_ stieg an und eine intermittierende nichtinvasive Beatmung wurde notwendig. Ohne Nachweis einer viralen oder bakteriellen Ursache bestand als wichtigste Differenzialdiagnose der Verdacht auf eine pulmonale Vaskulitis. Unter dem rasch progredienten Verlauf wurden eine Prednisolonstoßtherapie und Plasmapherese eingeleitet. Erst jetzt gab der Patient zu, regelmäßig Kokain zu schnupfen und „gelegentlich“ als sog. *Freebase* (selbstaufbereitet mit Ammoniak) oder als *Crack* zu inhalieren. Ohne den Nachweis von Autoantikörpern ergab sich die Diagnose einer toxischen Lungenschädigung. Im weiteren Verlauf musste der Patient intubiert und invasiv beatmet werden. Die antiinfektive Therapie wurde bei Nachweis von *Klebsiella oxytoca *auf Meropenem eskaliert. Der Patient wurde tracheotomiert und musste protrahiert geweant werden. Eine Rehabilitation erfolgte in einer neurologischen Rehabilitationsklinik. Auch nach 4 Wochen bestand weiterhin eine respiratorische Partialinsuffizienz mit Bedarf einer Sauerstofftherapie.

## Diagnose

In der Gesamtschau der Befunde lag eine sog. *„crack lung“* mit bipulmonalen Infiltraten, pulmonaler Hämorrhagie und bakterieller Superinfektion vor.

## Diskussion

In den 80er-Jahren kam es ausgehend von den Vereinigten Staaten im Rahmen des Kokainbooms zur Entwicklung von *Crack* als günstigere, inhalative Möglichkeit des Konsums. Kokain lässt sich durch die Zugabe von Natriumbicarbonat, enthalten in Backpulver, zu *Crack* oder alternativ durch Ammoniak zu *Freebase* weiterverarbeiten. Hierdurch entstehen kleine gelblich-weiße Kristallkörnchen, die durch Erhitzen inhalativ über die Lunge aufgenommen werden können. Hilfsmittel sind z. B. spezielle Crackpfeifen [1]. Durch das Erhitzen der Kristallkörnchen entsteht ein Knistern, das gemäß der englischen Übersetzung (*„to crackle“*) namensgebend für die Substanz ist, im Unterschied zu Kokain tritt die euphorisierende Wirkung von *Crack *schneller ein, da es innerhalb von wenigen Sekunden die Blut-Hirn-Schranke passiert, und hält nur wenige Minuten an [2]. In den vergangenen Jahren wird über einen steigenden *Crack*-Konsum in den deutschen Großstädten berichtet [3]. Gemäß dem Epidemiologischen Suchtsurvey (ESA) von 2021 liegt die Lebenszeitprävalenz für den Konsum von Kokain/*Crack* bei 5,6 %.

Auf pathophysiologischer Ebene kommt es durch die Inhalation von *Crack* zu einer Aktivierung des Sympathikotonus, zur Ruptur submuköser Atemwegsgefäße sowie zur durch Vasokonstriktion verursachten hypoxischen Schädigung der pulmonalen Gefäße. Infolgedessen kann es zum Anstoß einer Entzündungskaskade mit interstitiellem Lungenödem und alveolären Hämorrhagien kommen [4]. Diese Prozesse manifestieren sich in unterschiedlich schweren klinischen Erscheinungsbildern von lediglich trockenem Husten bis hin zum schweren ARDS mit pulmonalem Versagen unabhängig von der Konsumdauer und -frequenz. In dem hier berichteten Fall waren die Differenzialdiagnosen eine PcP bei radiologischem Muster mit Aussparung der Lungenperipherie und eine Vaskulitis [5]. Bei negativer Autoantikörperdiagnostik und ohne den Nachweis von *Pneumocystis jirovecii* (JP) in der Brochoalveolären Lavage (BAL) sowie bei zugegebenem regelhaftem Konsum von *Crack* und *Freebase* erschien nunmehr eine bipulmonale Schädigung im Rahmen einer *„crack lung“* als wahrscheinlichste Ausschlussdiagnose.

## Fazit für die Praxis

Der Konsum von aus Kokain hergestellten Substanzen ist in Deutschland verbreitet und das sogenannte *„crack lung syndrome“* sollte als Differenzialdiagnose beim Bild eines ARDS mit erwogen werden. Vor allen Dingen die Anamnese ist wichtig für die Diagnosestellung. Bezüglich der Therapie muss auf Begleitprobleme, wie eine Pneumonie, geachtet werden. Der Einsatz von oralen Kortikosteroiden wird kontrovers diskutiert. Bei Nachweis einer entzündlichen Komponente, wie einer eosinophilen Pneumonie, ist durchaus der Einsatz von Steroiden sinnvoll.
